# Anti-Tn Monoclonal Antibody Ameliorates Hyperoxia-Induced Kidney Injury by Suppressing Oxidative Stress and Inflammation in Neonatal Mice

**DOI:** 10.1155/2021/1180543

**Published:** 2021-10-21

**Authors:** Julie Chi Chow, Hsiu-Chu Chou, Jaulang Hwang, Chung-Ming Chen

**Affiliations:** ^1^Department of Pediatrics, Chi Mei Medical Center, Tainan, Taiwan; ^2^Department of Pediatrics, School of Medicine, College of Medicine, Taipei Medical University, Taipei, Taiwan; ^3^Department of Anatomy and Cell Biology, School of Medicine, College of Medicine, Taipei Medical University, Taipei, Taiwan; ^4^Taipei Cancer Center, Taipei Medical University, Taipei, Taiwan; ^5^Department of Pediatrics, Taipei Medical University Hospital, Taipei, Taiwan

## Abstract

The Tn antigen, an N-acetylgalactosamine structure linked to serine or threonine, has been shown to induce high-specificity, high-affinity anti-Tn antibodies in mice. Maternal immunization with the Tn vaccine increases serum anti-Tn antibody titers and attenuates hyperoxia-induced kidney injury in neonatal rats. However, immunizing mothers to treat neonatal kidney disease is clinically impractical. This study is aimed at determining whether anti-Tn monoclonal antibody treatment ameliorates hyperoxia-induced kidney injury in neonatal mice. Newborn BALB/c mice were exposed to room air (RA) or normobaric hyperoxia (85% O_2_) for 1 week. On postnatal days 2, 4, and 6, the mice were injected intraperitoneally with PBS alone or with anti-Tn monoclonal antibodies at 25 *μ*g/g body weight in 50 *μ*L phosphate-buffered saline (PBS). The mice were divided into four study groups: RA + PBS, RA + anti-Tn monoclonal antibody, O_2_ + PBS, and O_2_ + anti-Tn monoclonal antibody. The kidneys were excised for histology, oxidative stress, cytokine, and Western blot analyses on postnatal day 7. The O_2_ + PBS mice exhibited significantly higher kidney injury scores, 8-hydroxy-2'-deoxyguanosine (8-OHdG) and nuclear factor-*κ*B (NF-*κ*B) expression, and cytokine levels than did the RA + PBS mice or RA + anti-Tn mice. Anti-Tn monoclonal antibody treatment reduced kidney injury and cytokine levels to normoxic levels. The attenuation of kidney injury was accompanied by a reduction of oxidative stress and NF-*κ*B expression. Therefore, we propose that anti-Tn monoclonal antibody treatment ameliorates hyperoxia-induced kidney injury by suppressing oxidative stress and inflammation in neonatal mice.

## 1. Introduction

Supplemental oxygen is often required to treat newborns with respiratory disorders. However, administering high-concentration oxygen to newborn infants with respiratory failure increases oxidative stress and leads to kidney injury [1]. Prolonged hyperoxia induces glomerular and tubular damage in neonatal rodents, which manifests as enlarged renal corpuscles, renal tubular necrosis, interstitial inflammation, and kidney fibrosis during the perinatal period [2–6]. These harmful effects may continue into adulthood [4, 7]. Currently, no effective therapy is available for preventing the development of hyperoxia-induced kidney injury.

The Tn antigen is an N-acetylgalactosamine (GalNAc) residue that is *α*-linked to a serine or threonine residue, and it is one of the most notable tumor-associated carbohydrate antigens [8]. The Tn antigen is a pan-carcinoma antigen and is expressed in breast, pancreas, colon, lung, and bladder carcinomas and, less commonly, in hematological malignancies [9, 10]. Chiang et al. used linear array epitope technology to develop an anti-Tn vaccine that induces high-specificity, high-affinity anti-Tn antibodies in mice [11]. In our previous study, maternal immunization with the Tn vaccine increased serum levels of anti-Tn antibodies and reduced hyperoxia-induced kidney injury in neonatal rats by attenuating oxidative stress and nuclear factor-*κ*B (NF-*κ*B) activity [12]. These findings suggest that anti-Tn antibody treatment has therapeutic effects for hyperoxia-induced kidney injury. Hence, we hypothesized that anti-Tn monoclonal antibody treatment could attenuate hyperoxia-induced kidney injury in neonatal mice. The present study evaluated whether anti-Tn monoclonal antibodies can protect against hyperoxia-induced kidney injury and examined the mechanisms underlying these protective effects.

## 2. Materials and Methods

### 2.1. Animal Model

All experimental procedures were approved by the Animal Care and Use Committee of Taipei Medical University and were performed in accordance with institutional guidelines. Timed-pregnant BALB/c mice delivered pups vaginally at term. Within 12 h of birth, litters were pooled, randomly redistributed to the newly delivered mothers, and then exposed to either hyperoxia (O_2_) or room air (RA). The nursing mothers were rotated between O_2_ treatment and RA control litters every 24 h to avoid oxygen toxicity to the mothers and eliminate maternal effects between the treatment groups. An oxygen-rich atmosphere was maintained in a transparent 40 × 50 × 60 cm^3^ plexiglass chamber that received O_2_ continuously at 4 L/min. Oxygen levels were monitored using a ProOx Model 110 monitor (NexBiOxy, Hsinchu, Taiwan). The hyperoxia groups were placed in an environment with 85% O_2_ for 1 week, and the RA groups were kept in a normoxic environment for 1 week. Anti-Tn monoclonal antibodies were generated and administered as described previously [13]. The anti-Tn monoclonal antibody-treated groups were intraperitoneally injected with anti-Tn monoclonal antibodies at 25 *μ*g/g body weight in 50 *μ*L phosphate-buffered saline (PBS) on postnatal days 2, 4, and 6. The dosage was not evaluated for kidney injury in the murine pups exposed to postnatal hyperoxia. We chose this dosage because it increased anti-Tn antibody serum levels 8~10 folds higher in treated group over PBS group on postnatal day 7 [13]. The PBS-treated groups were injected with equivalent amounts of PBS on the same days. The mice were divided into four study groups: RA + PBS, RA + anti-Tn monoclonal antibody, O_2_ + PBS, and O_2_ + anti-Tn monoclonal antibody. On postnatal day 7, mice pups were anesthetized with 1% isoflurane (Halocarbon Laboratories, River Edge, NJ, USA) and were weighed. The kidneys were excised for histological, Western blot, and cytokine analyses. The kidneys used for these experiments were obtained from a previous study designed to assess lung injury [13].

### 2.2. Histology

Kidneys were placed in 4% paraformaldehyde, washed in PBS, and serially dehydrated in increasing concentrations of ethanol and were then embedded in paraffin. Five-micrometer tissue sections were stained with hematoxylin and eosin (H&E) and periodic acid-Schiff (PAS), and semiquantitative analysis of kidney injury was performed as described previously [14].The following parameters were used to grade the extent of kidney injury: (1) glomerular injury (% of renal parenchyma involvement): none = 0, <25% of glomeruli exhibiting nonspecific features of injury = +1, 25%–50% of glomeruli exhibiting nonspecific features of injury = +2, 50%–75% of glomeruli exhibiting nonspecific features of injury = +3, and >75% of glomeruli exhibiting nonspecific features of injury = +4; (2) acute tubular necrosis (% of renal parenchyma involvement): none = 0, <25% of tubules out of the entire renal parenchyma = +1, 25%–50% of tubules out of the entire renal parenchyma = +2, 50%–75% of tubules out of the entire renal parenchyma = +3, and >75% of tubules out of the entire renal parenchyma = +4; and (3) tubulointerstitial inflammatory infiltrates: none = 0, leukocytes confined within the interstitium = +1, and leukocytes infiltrating the interstitium and tubular epithelial cells = +2.

We have examined and graded predominant injury patterns including tubular injury and glomerular damage. Tubular injury included tubular dilation, tubular atrophy, vacuolization, the degeneration and sloughing of tubular epithelial cells, or thickening of the tubular basement membrane. All slides were examined by light microscope (×200 magnification), and tubular injury was evaluated semiquantatively on 5 nonoverlapping fields per slide. The renal tubules were used in the scoring system, where 0 = no tubular injury; 1 = <10% of tubules injured; 2 = 10-25% of tubules injured; 3 = 26-50% of tubules injured; 4 = 51-75% of tubules injured; and 5 = >75% of tubules injured [15]. The degree of mesangial matrix expansion within the glomerular tuft was determined on PAS stained paraffin sections. A glomerular score for each animal was derived from the examination of 20 glomeruli per slide using light microscopy at a magnification of 400x. The glomerular score was grade 0, normal glomeruli; grade 1, presence of mesangial expansion/thickening of the basement membrane; grade 2, mild/moderate segmental hyalinosis/sclerosis involving less than 50% of the glomerular tuft; grade 3, diffuse glomerular hyalinosis/sclerosis involving >50% of the tuft; grade 4, diffuse glomerulosclerosis with total tuft obliteration and collapse [16].

### 2.3. Western Blot Analysis of NF-*κ*B and I*κ*B-*α*

We extracted nuclear proteins to detect NF-*κ*B p65 (Santa Cruz Biotechnology, CA, USA). Protein concentrations were determined using a bicinchoninic acid protein assay kit. After membranes were blocked with 5% skim milk at room temperature for 1 h, proteins were separated on a 12% sodium dodecyl sulfate polyacrylamide gel and were transferred onto polyvinylidene difluoride membranes. The membranes were incubated with HRP-conjugated secondary antibodies at room temperature for 1 h. Protein bands were visualized using enhanced chemiluminescence reagents according to manufacturer's protocol. Antibodies to *β*-actin were used as internal controls for nuclear protein loading.

### 2.4. Immunohistochemistry

Immunohistochemical staining of 5 *μ*m paraffin sections was performed using immunoperoxidase visualization. After routine deparaffinization, heat-induced epitope retrieval was performed by immersing the slides in 0.01 M sodium citrate buffer (pH 6.0). The sections were then incubated for 20 h at 4°C with mouse monoclonal anti-8-hydroxy-2'-deoxyguanosine (8-OHdG) antibodies (1 : 500; Abcam Inc., Cambridge, MA, USA) and anti-NF-*κ*B-p65 (F-6) and anti-inhibitor of *κ*B (I*κ*B-*α*) antibodies (H-4) (1 : 500; Santa Cruz Biotechnology, Inc., CA, USA) as primary antibodies. The sections were washed in PBS and then treated for 1 h at 37°C with biotinylated goat anti-mouse IgG (1 : 5000, Jackson ImmunoResesarch Laboratories Inc., PA, USA). After incubation with biotinylated IgG, the sections were reacted with reagents from an ABC kit (Avidin-Biotin Complex, Vector). To obtain the end reaction products, the sections were reacted with reagents of the diaminobenzidine substrate kit (Vector Laboratories, Inc., Burlingame, CA, USA) according to manufacturer's recommendations. All the immunostained sections were viewed and photographed using a Nikon Eclipse E600. Positive 8-OHdG cell nuclei were scored in five randomly selected fields from each section at 400x magnification, and images were captured using a digital camera and imported into a computerized image analysis system (Image-Pro Plus 5.1 for Windows). This provided a percentage of positively stained cells; values are expressed as a labeling index (%).

### 2.5. Cytokines

Kidney interleukin (IL)-6 and IL-1ß levels were determined using the Bio-Plex multiplex assay system (Bio-Rad, Hercules, CA, USA) and the Procarta immunoassay kit (Affymetrix), according to manufacturer's protocol.

### 2.6. Statistical Analysis

All data are presented as mean ± SD. Data were compared using two-way analysis of variance (ANOVA) and the Bonferroni post hoc test. A *P* value of <0.05 was considered statistically significant.

## 3. Results

### 3.1. Anti-Tn Monoclonal Antibodies Improved Hyperoxia-Induced Kidney Injury

**Figure 1:**
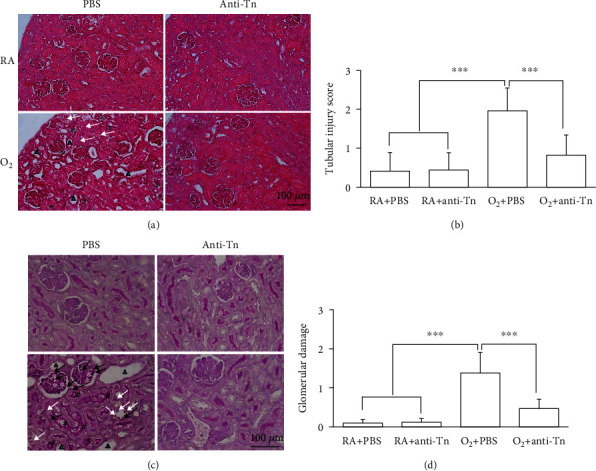
Representative kidney sections stained with (a) hematoxylin and eosin, (b) PAS, and (c) tubular injury score and (d) glomerular damage from the RA-reared and hyperoxia-reared mice treated with PBS or anti-Tn monoclonal antibodies on postnatal day 7. The mice reared in RA and treated with PBS or anti-Tn monoclonal antibodies exhibited no glomerular injury, acute tubular necrosis, or tubulointerstitial inflammatory infiltrates. The mice reared in hyperoxia and treated with PBS exhibited tubular atrophy, dilatation of the tubular lumen (triangles), degenerated tubular cells (white arrows), increased space between the renal tubules (black asterisks), distortion of the free surface of tubular cells (hashtags), thickened basement membrane of glomerular capillaries and renal tubules (black arrows), and mesangial expansion between the glomerular capillaries (white asterisks). Anti-Tn monoclonal antibody treatment improved hyperoxia-induced kidney injury. ^∗∗∗^*P* < 0.001.

Representative kidney sections stained with H&E and PAS from each treatment group are shown in Figure 1. The histological changes for all experimental groups are summarized in Table 1. The RA + PBS and RA + anti-Tn mice exhibited no glomerular injury, acute tubular necrosis, or tubulointerstitial inflammatory infiltrates. The O_2_ + PBS mice exhibited significantly higher kidney injury scores than the RA + PBS and RA + anti-Tn mice. Treatment with anti-Tn monoclonal antibodies significantly decreased the hyperoxia-induced increase in kidney injury scores.

The RA + PBS and RA + anti-Tn mice displayed a normal kidney structure, with no evidence of tissue injury (Figure 1(a)). Tubular atrophy, dilatation of the tubular lumen, degenerated tubular cells, and increased space between the renal tubules were observed in the O_2_ + PBS mice. The O_2_ + PBS mice exhibited significantly higher tubular injury score than did the RA + PBS and RA + anti-Tn mice, and anti-Tn monoclonal antibody treatment reduced the hyperoxia-induced increase in tubular injury score (Figure 1(b)). We used PAS staining to evaluate polysaccharide accumulation in microvilli, basement membranes, and mesangium (Figure 1(c)). The RA + PBS and RA + anti-Tn mice exhibited a relatively intact brush border structure, and the O_2_ + PBS mice exhibited a distorted free surface of tubular cells. Moreover, the O_2_ + PBS mice displayed thickened basement membranes in glomerular capillaries and renal tubules as well as markedly expanded mesangium between the glomerular capillaries. The O_2_ + PBS mice exhibited significantly higher glomerular damage score than did the RA + PBS and RA + anti-Tn mice, and anti-Tn monoclonal antibody treatment reduced the hyperoxia-induced increase in glomerular damage score (Figure 1(d)).

### 3.2. Anti-Tn Monoclonal Antibody Reduced Renal Oxidative Stress

**Figure 2:**
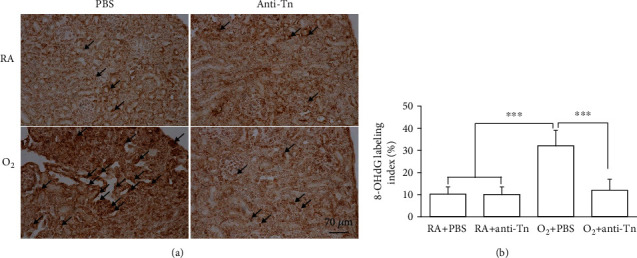
(a) Representative immunohistochemistry of 8-OHdG and (b) semiquantitative analysis of 8-OHdG-positive cells on postnatal day 7. The mice reared in hyperoxia and treated with PBS exhibited significantly more 8-OHdG-positive cells (black arrow) than did the RA-reared mice treated with PBS or anti-Tn monoclonal antibodies, and anti-Tn monoclonal antibody treatment reduced the hyperoxia-induced increase in 8-OHdG-positive cells. ^∗∗∗^*P* < 0.001.

The oxidative stress marker 8-OHdG was positively stained in the glomerular and tubular cell nuclei (Figure 2(a)). The O_2_ + PBS mice exhibited significantly more 8-OHdG-positive cells than did the RA + PBS and RA + anti-Tn mice, and anti-Tn monoclonal antibody treatment reduced the hyperoxia-induced increase in 8-OHdG-positive cells (Figure 2(b)).

### 3.3. Immunohistochemistry and Western Blotting for NF-*κ*B-p65 and I*κ*B-*α*

**Figure 3:**
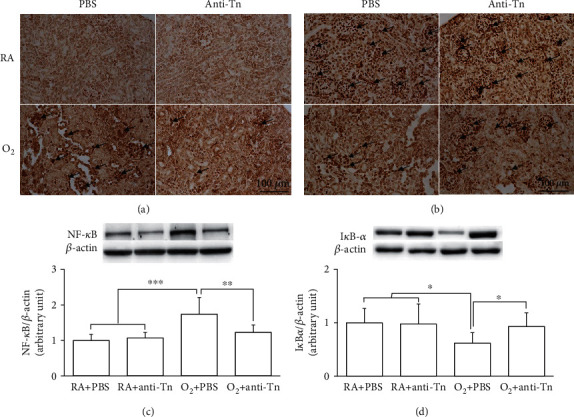
(a, c) Representative immunohistochemistry and (b, d) Western blot analysis of NF-*κ*B and I*κ*B-*α* in the RA-reared and hyperoxia-reared rats treated with PBS or anti-Tn monoclonal antibodies on postnatal day 7. The immunoreactivity of NF-*κ*B-p65 (black arrow) and I*κ*B-*α* (black arrow) was primarily detected in the podocytes of the glomerulus and the tubular cells of kidney tissue. The mice reared in hyperoxia and treated with PBS exhibited a higher number of positive NF-*κ*B-p65 and fewer positive I*κ*B-*α* cells. Anti-Tn monoclonal antibody treatment significantly reduced the hyperoxia-induced increase in NF-*κ*B expression and increased the hyperoxia-induced decrease in I*κ*B-*α* expression. ^∗^*P* < 0.05, ^∗∗∗^*P* < 0.001.

NF-*κ*B-p65 and I*κ*B-*α* protein expression in the kidney tissue was detected using immunohistochemical assays. The immunoreactivity of NF-*κ*B-p65 and I*κ*B-*α* was primarily detected in the podocytes of the glomerulus and the tubular cells of the kidney tissue (Figures 3(a) and 3(c)). No obvious NF-*κ*B immunoreactivity was observed in the RA + PBS or RA + anti-Tn mice. The O_2_ + PBS mice exhibited a high number of NF-*κ*B-p65-positive cells and fewer I*κ*B-*α*-positive cells. Anti-Tn monoclonal antibody treatment significantly reduced the hyperoxia-induced increase in NF-*κ*B immunoreactivity and enhanced the hyperoxia-induced decrease in I*κ*B-*α* immunoreactivity.

### 3.4. Anti-Tn Monoclonal Antibodies Decreased Hyperoxia-Induced Increase in Cytokines

**Figure 4:**
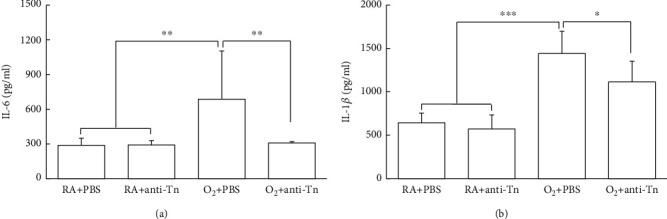
Anti-Tn monoclonal antibody treatment reduced the hyperoxia-induced increase in cytokine levels in 7-day-old mice. The mice reared in hyperoxia and treated with PBS exhibited significantly higher IL-6 and IL-1*β* than did those reared in RA and treated with PBS or anti-Tn monoclonal antibodies. Treatment with anti-Tn monoclonal antibodies significantly decreased the hyperoxia-induced increase in IL-6 and IL-1*β* levels. ^∗^*P* < 0.05, ^∗∗^*P* < 0.01, ^∗∗∗^*P* < 0.001.

The O_2_ + PBS mice exhibited significantly higher IL-6 and IL-1*β* levels than did the RA + PBS or RA + anti-Tn mice (Figures 4(a) and 4(b)). Furthermore, anti-Tn monoclonal antibody treatment reduced the hyperoxia-induced increase in IL-1*β* and IL-6 levels.

## 4. Discussion

Our *in vivo* model demonstrated that hyperoxia exposure during the first 7 days after birth induced kidney injury, as demonstrated by increased kidney injury scores and cytokine levels. Anti-Tn monoclonal antibody treatment reversed the hyperoxia-induced increase in kidney injury scores and cytokine levels, suggesting that anti-Tn antibodies may ameliorate hyperoxia-induced kidney injury. The improvement of kidney injury was associated with a reduction in oxidative stress, NF-*κ*B expression, and cytokine levels. Therefore, we propose that anti-Tn monoclonal antibodies improve hyperoxia-induced kidney injury in neonatal mice by suppressing oxidative stress and inflammation. These findings suggest that anti-Tn antibodies are a candidate treatment strategy for hyperoxia-induced kidney injury.

Kidney development is completed *in utero* by gestational week 36 in human fetuses [17]. The nephrogenesis of rodents begins on embryonic day 12 and is completed between 10 and 15 days after birth [18]. Rodents are born with immature kidneys, and the first 2 postnatal weeks correspond to the second and third trimesters of kidney development in the human fetus. Thus, the neonatal mouse model is suitable for studying the effects of hyperoxia on kidney development in the human fetus.

In a previous study, we found that maternal immunization with the Tn vaccine attenuated hyperoxia-induced kidney injury and fibrosis in newborn rats exposed to hyperoxia in the first 14 days of life [12]. Nevertheless, immunizing mothers to treat neonatal kidney diseases is not feasible because neonatal diseases are clinically unpredictable before birth. In this study, we evaluated the direct therapeutic effects of anti-Tn monoclonal antibodies on hyperoxia-induced kidney injury in neonatal mice. We demonstrated that hyperoxia-induced kidney injury increased oxidative stress and inflammation and that anti-Tn monoclonal antibodies improved hyperoxia-induced kidney injury in neonatal mice. This treatment strategy is feasible for clinical application.

Renal cells, including mesangial and tubular epithelial cells, express toll-like receptors (TLRs) and produce proinflammatory cytokines and chemokines that contribute to kidney injury [19]. Prolonged exposure to hyperoxia increases TLR and cytokine expression and induces inflammation in the kidneys of neonatal rats and mice [5, 20–22]. The I*κ*B protein inactivates NF-*κ*B dimers. The increase in TLR ligands and proinflammatory cytokines stimulates the phosphorylation-dependent I*κ*B complex and activates NF-*κ*B dimers [23]. In this study, hyperoxia-reared mice exhibited a significant increase in NF-*κ*B expression and a significant decrease in I*κ*B-*α* expression, and these changes were reversed by anti-Tn monoclonal antibody treatment. Anti-Tn antibody treatment reduced NF-*κ*B activity, which was associated with reduced levels of kidney cytokines. Our findings related to NF-*κ*B activity are consistent with previous knowledge of the role played by NF-*κ*B in kidney injury [23].

In conclusion, this study demonstrated that anti-Tn monoclonal antibody treatment ameliorated hyperoxia-induced kidney injury in neonatal mice, as indicated by decreased kidney injury scores and cytokine levels. The results of this study also indicated that anti-Tn monoclonal antibody treatment does not have adverse effects on normal neonatal kidney development. The advantageous effects of anti-Tn monoclonal antibodies on hyperoxia-induced kidney injury are mediated by a decrease in oxidative stress and NF-*κ*B expression as well as an increase in I*κ*B-*α* expression. Presently, no effective treatment is available for hyperoxia-induced kidney injury. Our findings suggest the therapeutic potential of anti-Tn monoclonal antibodies for hyperoxia-induced kidney injury.

## Figures and Tables

**Table 1:** Kidney injury scores of histological changes in the mice.

	Glomerular injury	Acute tubular necrosis	Tubulointerstitial inflammatory infiltrates	Total score
RA + PBS	0 (0-1)	0 (0-1)	0 (0-1)	1 (0-3)
RA + anti-Tn	0 (0-1)	0 (0-1)	0 (0-1)	1 (0-3)
Hyperoxia + PBS	2 (1-2)^a^	2 (1-2)^a^	1 (0-2)^a^	5 (2-6)^a^
Hyperoxia + anti-Tn	0 (0-1)^b^	0 (0-1)^b^	0 (0-1)^c^	2 (0-3)^b^

Data are presented as median (range). ^a^*P* < 0.001 vs. RA + PBS and RA + anti-Tn groups; ^b^*P* < 0.001 vs. hyperoxia + PBS group; ^c^*P* < 0.01 vs. hyperoxia + PBS group.

## Data Availability

The data used to support the findings of this study are available from the corresponding author upon request.

## References

[1] Stritzke A., Thomas S., Amin H., Fusch C., Lodha A. (2017). Renal consequences of preterm birth. *Molecular and Cellular Pediatrics*.

[2] Perrone S., Mussap M., Longini M. (2007). Oxidative kidney damage in preterm newborns during perinatal period. *Clinical Biochemistry*.

[3] Popescu C. R., Sutherland M. R., Cloutier A. (2013). Hyperoxia exposure impairs nephrogenesis in the neonatal rat: role of HIF-1*α*. *PLoS One*.

[4] Sutherland M. R., O'Reilly M., Kenna K. (2013). Neonatal hyperoxia: effects on nephrogenesis and long-term glomerular structure. *American Journal of Physiology-Renal Physiology*.

[5] Chen C. M., Chou H. C. (2019). Maternal inflammation exacerbates neonatal hyperoxia-induced kidney injury in rat offspring. *Pediatric Research*.

[6] Jiang J. S., Chou H. C., Yeh T. F., Chen C. M. (2015). Neonatal hyperoxia exposure induces kidney fibrosis in rats. *Pediatrics & Neonatology*.

[7] Yzydorczyk C., Comte B., Cambonie G. (2008). Neonatal oxygen exposure in rats leads to cardiovascular and renal alterations in adulthood. *Hypertension*.

[8] Zhang X., Issagholian A., Berg E. A., Fishman J. B., Nesburn A. B., BenMohamed L. (2005). Th-cytotoxic T-lymphocyte chimeric epitopes extended by Nepsilon-palmitoyl lysines induce herpes simplex virus type 1-specific effector CD8+ Tc1 responses and protect against ocular infection. *Journal of Virology*.

[9] Liu D. Y., Jiang T., Wang S., Cao X. (2013). Effect of hyperoxia on pulmonary SIgA and its components, IgA and SC. *Journal of Clinical Immunology*.

[10] Cheon I. S., Son Y. M., Jiang L. (2018). Neonatal hyperoxia promotes asthma-like features through IL-33-dependent ILC2 responses. *Journal of Allergy and Clinical Immunology*.

[11] Chiang H. L., Lin C. Y., Jan F. D. (2012). A novel synthetic bipartite carrier protein for developing glycotope-based vaccines. *Vaccine*.

[12] Chen C. M., Hwang J., Chou H. C. (2021). Immunization with anti-Tn immunogen in maternal rats protects against hyperoxia-induced kidney injury in newborn offspring. *Pediatric Research*.

[13] Chen C. M., Hwang J., Chou H. C., Chen C. (2020). Anti-Tn monoclonal antibody attenuates hyperoxia-induced lung injury by inhibiting oxidative stress and inflammation in neonatal mice. *Frontiers in Pharmacology*.

[14] Kader C., Sunbul M., Das Y. K. (2017). Telbivudine attenuates gentamicin-induced kidney injury in rats. *International Journal of Antimicrobial Agents*.

[15] Kuruş M., Ugras M., Esrefoglu M. (2009). Effect of resveratrol on tubular damage and interstitial fibrosis in kidneys of rats exposed to cigarette smoke. *Toxicology and Industrial Health*.

[16] el Nahas A. M., Bassett A. H., Cope G. H., Le Carpentier J. E. (1991). Role of growth hormone in the development of experimental renal scarring. *Kidney International*.

[17] Zoetis T., Hurtt M. E. (2003). Species comparison of anatomical and functional renal development. *Birth Defects Research Part B: Developmental and Reproductive Toxicology*.

[18] Seely J. C. (2017). A brief review of kidney development, maturation, developmental abnormalities, and drug toxicity: juvenile animal relevancy. *Journal of Toxicologic Pathology*.

[19] Anders H. J., Schlondorff D. (2007). Toll-like receptors: emerging concepts in kidney disease. *Current Opinion in Nephrology and Hypertension*.

[20] Chou H. C., Chen C. M. (2019). Cathelicidin attenuates hyperoxia-induced kidney injury in newborn rats. *Renal Failure*.

[21] Xu X., Zhang X., Gao L., Liu C., You K. (2020). Neonatal hyperoxia downregulates claudin-4, occludin, and ZO-1 expression in rat kidney accompanied by impaired proximal tubular development. *Oxidative Medicine and Cellular Longevity*.

[22] Yen C. C., Chang W. H., Tung M. C. (2020). Lactoferrin protects hyperoxia-induced lung and kidney systemic inflammation in an *in vivo* imaging model of NF-*κ*B/Luciferase transgenic mice. *Molecular Imaging and Biology*.

[23] Song N., Thaiss F., Guo L. (2019). NF*κ*B and kidney injury. *Frontiers in Immunology*.

